# The Integrative Method Based on the Module-Network for Identifying Driver Genes in Cancer Subtypes

**DOI:** 10.3390/molecules23020183

**Published:** 2018-01-24

**Authors:** Xinguo Lu, Xing Li, Ping Liu, Xin Qian, Qiumai Miao, Shaoliang Peng

**Affiliations:** 1 College of Computer Science and Electronic Engineering, Hunan University, Changsha 410082, China; xingleo@hnu.edu.cn (X.L.); qianxin@hnu.edu.cn (X.Q.); qiumaimiao@hnu.edu.cn (Q.M.); 2 Hunan Want Want Hospital, Changsha 410006, China; lp-simple123@126.com; 3 School of Computer Science, National University of Defense Technology, Changsha 410073, China

**Keywords:** integrative analysis, module network, cancer subtypes, breast cancer, copy number variation, gene expression

## Abstract

With advances in next-generation sequencing(NGS) technologies, a large number of multiple types of high-throughput genomics data are available. A great challenge in exploring cancer progression is to identify the driver genes from the variant genes by analyzing and integrating multi-types genomics data. Breast cancer is known as a heterogeneous disease. The identification of subtype-specific driver genes is critical to guide the diagnosis, assessment of prognosis and treatment of breast cancer. We developed an integrated frame based on gene expression profiles and copy number variation (CNV) data to identify breast cancer subtype-specific driver genes. In this frame, we employed statistical machine-learning method to select gene subsets and utilized an module-network analysis method to identify potential candidate driver genes. The final subtype-specific driver genes were acquired by paired-wise comparison in subtypes. To validate specificity of the driver genes, the gene expression data of these genes were applied to classify the patient samples with 10-fold cross validation and the enrichment analysis were also conducted on the identified driver genes. The experimental results show that the proposed integrative method can identify the potential driver genes and the classifier with these genes acquired better performance than with genes identified by other methods.

## 1. Introduction

Breast cancer is one of the most common malignant tumors in women. The incidence rate is 7–10% of all kinds malignant tumors which is usually associated with genetic alterations [1]. Breast cancer has been categorized into five subtypes, including luminal A (LumA), luminal B (LumB), HER2-enriched (HER2), basal-like (Basal), and normal-like (Normal) types. Previous studies have shown that each cancer subtype has its own gene imprint and tumor markers, and genetic variation will increase the risk of cancer. However, not all of the aberrations have the same impact on tumor progression. To understand the mechanism of cancer, identifying driver genes from genomic aberrations has become the focus of research. Meanwhile, the gene expression profiles play an important role in understanding the pathogenesis of disease. The gene expression profiles provide information about their activity level. Activation or deactivation of the functional parts of a genome, or genes, determines the pathological states and development of a disease. The over-expression of an oncogene or under-expression of a tumor suppressor gene also has a certain influence on cancer process. So it is reasonable to believe that an over/under-expressed gene has a footprint in a genome in the form of an aberration that can be used as a biomarker [2,3,4]. Besides, with the availability of huge amounts of multiple genomics data, the comprehensive analysis across the different genomics data will improve the understanding of the role of biomarkers in breast cancer pathogenesis and procession [5].

With the development of high-throughput sequencing technologies, huge volumes of diseased-based histological data have been provided in life science research. These data are publicly available in databases, such as The Cancer Genome Atlas (TCGA) and International Cancer Genome Consortium (ICGC) in which several high throughput genomic data types for hundreds of sample on tens of cancer types have generated. Recently, many methods which integrated multi-types of genomic data have been developed to reveal combinatorial patterns and discover new biological mechanisms [6]. Zhang et al. developed an unbiased adaptive clustering approach to integrate and analyze the multi-types of genomics data for ovarian cancer, including genome-wide gene expression, DNA methylation, microRNA expression, and copy number alteration profiles. And they developed an algorithm to uncover molecular signatures that distinguish cancer subtypes [7]. Huang et al. proposed a multiple regression based method to construct an integrative network with gene expression, microRNA, methylation and copy number variation [8]. In addition, to explore the important role of genomics aberrations and gene expression profiles in disease progression, some studies have also committed to discover novel candidate driver genes by integrating gene expression and CNV data. Li et al. identified the breast cancer subtype-specific drivers by integrating and analyzing the copy number aberrations data and miRNA-mRNA dual expression profiling data [9]. However, these proposed methods are based on linear models, such as regression analysis and correlation analysis, which is not suitable for heterogeneous data that have extremely high within-group variations. As we know that there is a significant heterogeneity in breast cancer data. We expect this limitation of linear approaches can be solved for the heterogeneous data.

To this end, we present a novel computational framework using module network analysis [10] to identify the breast cancer subtype-specific driver genes by integrating the gene expression and CNV data. In this framework, we first selected the subsets of gene expression and CNV data with the differential analysis. Then, a module network is constructed as a form of Bayesian network in which the similarly behaving variables are clustered into modules and the same parents and parameters are learned for each module, instead of each variable. A module is defined as a set of co-expressed or co-regulated genes that share a common statistical model. Through the process of module network learning, we obtained the candidate drivers of each subtype. The final subtype-specific driver genes were acquired by comparing paired-wise subtypes. The classification algorithm is used to validate whether subtype-specific driver genes can distinguish subtypes as well. To better understand the underlying biological significance, the pathway impact analysis and functional analysis is applied to explicate regulation mechanism of these driver genes. The results have shown that the proposed method is able to detect highly mutated gene as subtype-specific driver genes and the identified driver genes can classify breast cancer subtypes as well.

## 2. Results

We downloaded the clinical records and five breast cancer subtypes of high-throughput data consisting of initial 825 patients from TCGA. To increase to the statistic power, we take a filtering strategy to ensure that each sample both has gene expression and CNV data for analysis. And this process resulted in 485 samples. For each sample, the gene expression data includes expression level of 17,268 genes and the CNV values of 20,871 genes is obtained.

Our experiment is constituted by three parts: (1) identifying the subtype-specific driver genes using the proposed integrative method; (2) comparing the classification performance. The classifiers are constructed by these driver genes. And the performance is compared with the information gain, Chi-squared and lemon-tree methods; (3) analyzing biological significance of the obtained driver genes, including topology-based pathway analysis, Gene ontology (GO) functional enrichment, KEGG pathway enrichment analysis.

For gene expression and CNV data, the difference between each subtype and other three subtypes was analyzed. In gene expression data, we selected genes with *q*-values < 0.1 which represented the gene was differentially expressed between subtypes. And for CNV data, we firstly selected genes with their *q*-values < 0.1. Then we calculated the frequency of amplification and deletion for each gene in each of the two subtypes samples and selected genes for which the difference of frequencies between two subtypes is more than 20%. Additionally, we used a threshold of log2 copy number variation ratio of 0.3/−0.3 to call amplified/deleted genes. And the gene subsets are converged and the relevant subset of data is selected for each subtype. We selected the genes as candidate modulators with mutation frequency fre > 0.6. We compiled a list of 965 candidate modulators for the HER2, 336 candidate modulators for lumA, 1213 candidate modulators for lumB, 815 candidate modulators for basal.

Through data preprocessing above, module-network analysis is used to select candidate driver genes. The whole learning algorithm is performed 100 times. We filtered the set of candidate modulators and left only genes that appeared in at least one regulation program in at least 40% of the runs. These modulators are considered as candidate driver genes. Thus, the procedure resulted in 148 modules with 9 candidate drivers for HER2 samples, 550 modules with 28 candidate drivers for lumA samples, 258 modules with 12 candidate drivers for lumB samples, 201 modules with 14 candidate drivers for basal samples. Several modules shared the same modulators in each subtypes of samples. Interestingly, the result of module network analysis for HER2 and other three subtypes represented that they shared three genes. Finally, the candidate driver genes in this subtype, but not those in other subtypes, are the subtype-specific driver genes, including 8 Her2 drivers, 11 Basal drivers, 28 LumA drivers and 10 LumB drivers.

**Figure 1:**
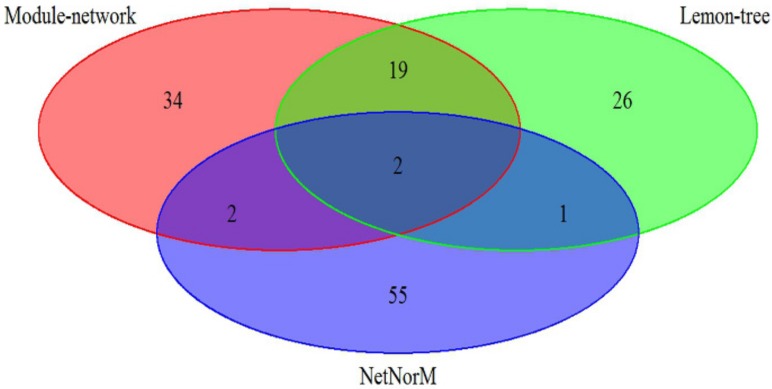
A comparison of identified cancer drivers between module-network and two state of the integrative methods on breast cancer subtypes datasets.

The effectiveness of our approach was compared against two other state-of-art model-based algorithm, Lemon-tree [11] and NetNorM [12], in which the breast cancer datasets is utilize to analyze. As shown in Figure 1, the 57 driver genes are identified by Module-network, while Lemon-tree and NetNorM identified 48 and 60 drivers respectively. There are two driver genes identified by three methods, ATP1A2 and IFI16 respectively. In addition, the 21 drivers were identified by methods of Module-network and Lemon-tree together, and only 4 driver genes are selected by methods of Module-network and NerNorM together. This may be because NetNorM method is based on the mutation data and gene-gene interaction data, while the other two methods integrated the mutation data and gene expression data. The results suggest that the integrative approach can recognize the driver genes for each subtypes.

### 2.1. Identified Novel Subtype-Specific Driver Genes in LumA

**Figure 2:**
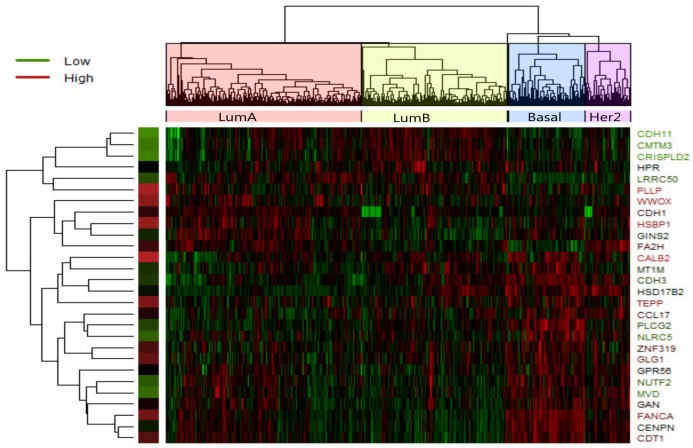
Heatmap of expression values of 28 most significant differently expressed gene in LumA-subtype. Clustering method on expression values was used to generate the heatmap. There are clear clusters of genes for the four tumor subtypes.

In LumA , we identified 28 candidate driver genes which are frequently mutated. A heatmap of the 28 significantly differentially expressed genes of LumA is shown in Figure 2 which represented that expression profiles can be clearly clustered into four subtypes by using the selected driver genes. In addition, we found that some samples were mixed between the LumA and the LumB in the process of clustering. We supposed that LumA and LumB belong to the luminal, and luminal subtype has the lowest overall mutation rate.

**Figure 3:**
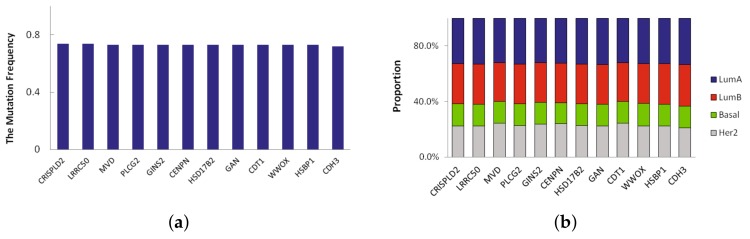
(**a**) The top 12 mutation frequency of driver genes in LumA; (**b**) The mutation proportion of the top 12 genes in each breast cancer subtype samples.

A novel significant driver of the subtype is a frequently mutated gene in specific subtype that has not been classified as a driver gene. Here we defined novel significant driver genes which satisfy the following requirements: (1) subtype-specific driver genes recurrently mutated in multiple subtypes. The mutation frequency of the driver genes is different from other three subtypes, and their difference is more than 0.2; (2) not previously classified as a driver by CGC database [13]. In LumA, we found 23 novel significant driver genes which recursively mutated. We sorted them according to mutation frequency and the mutation frequency of top 12 driver genes in LumA_subtype is shown in Figure 3a. The mutation frequency of 12 driver genes identified in LumA is more than 0.7. Moreover, the proportion of mutation samples in each breast cancer subtype was shown in Figure 3b. The proportion of LumA is obviously higher than the other three cancer subtypes.

Of the 23 driver genes, CDH3, GLG1, CCL17 and PLCG2 are the most promising. These genes are significantly mutated and are involved in several cancer functions and pathways. CDH3 has the highest mutation frequency and is mutated in 72% of LumA samples. CDH3 belongs to the family of classic cadherins that are engaged in various cellular activities including motility, invasion, and signaling of tumor cells, in addition to cell adhesion [14]. GLG1 is a key ligand-receptor in the early response of cells [15]. The mutation frequency of CCL17 in LumA samples is 0.7. CCL17 is a central regulator of Treg homeostasis, and CCL17 might be a target for vascular therapy [16]. A CCL17 gene is a candidate as one of the genetic factors in some allergic diseases [17]. PLCG2 encodes phospholipase C${}_{Y2}$(PLC${}_{Y2}$), an enzyme with a critical regulatory role in various immune and inflammatory pathways, and plays a key role in the regulation of immune response [18]. PLAID-associated deletions of PLCG2 cause diminished receptor-mediated activity at physiologic temperatures in B cells and natural killer cells with enhanced spontaneous signaling in mast cells and B cells at subphysiologic temperatures [19].

### 2.2. Identified Novel Subtype-Specific Driver Genes in Basal/LumB/Her2

Similarly, we can also identify the subtype-specific driver genes of other three subtypes. In Basal, there are several novel significant driver genes and two of these are strong novel drivers: FDPS and ATP1A2. FDPS is a key enzyme in the isoprenoid pathway responsible for cholesterol biosynthesis, post-translational protein modifications and synthesis of steroid hormones, whose expression is regulated by phorbol esters and polyunsaturated fatty acids [20]. FDPS is necessary for osteoclast survival and activity and is considered as a major molecular target of aminobisphosphonates [21]. The ATP1A2 gene encodes the α2 subunit of Na${}^{+}$-, K${}^{+}$-ATPase, a plasma membrane enzyme that counter transports Na${}^{+}$ and K${}^{+}$ across cell membranes [22]. ATP1A2 gene mutation result in degeneration of the amygdala and pyriform cortex [23].

In LumB, two potential novel drivers is FASLG and RGS2. Alteration of FASLG pathway regulating cell death may lead to cancer development [24]. Studies have revealed that increased FASLG expression facilitate development and progression of tumors, including gastric cancer. These results suggest that variants of the FASLG gene is likely to be associated with the initiation and development of gastric cancer [25]. The mutation frequency of FASLG is about 80% in LumB. RGS2 is a member of a family of proteins that negatively modulate G-protein coupled receptor transmission. Variations in the RGS2 gene were found to be associated in humans with anxious and depressive phenotypes [26]. RGS2 is mutated in 78% Basal samples.

In Her2, there are fewer candidate driver genes than Luma, however, we detected two genes that have powerful potential to become novel drivers: ZFPM2 and EGLN1. ZFPM2 protein is an important cofactor for GATA family of transcription factors. In adult tissues, the ZFPM2 protein of 1151 amino acids is expressed predominantly in brain, heart, and testis. ZFPM2 may act as repressor or activator, depending on specific promoter and cell type [27]. EGLN1 is a key oxygen sensor gene that negatively regulates the activity of hypoxia-in-ducible factor (HIF-1A). Owing to its important function as an oxygen sensor, EGLN1 is relevant to the human hypoxic response, both at high altitude in hypoxic conditions or in cellular hypoxia [28]. EGLN1 is an important gene functioning at the upstream of the HIF pathway and showed consistent selective signals across multiple studies [29].

### 2.3. Validation of Classification between Subtypes

To validate whether our subtype-specific driver genes obtained from integration analysis are also applicable to distinguish subtypes, we classified the samples between subtypes using SVM [30]. SVM is a linear maximum-margin model for classification [31]. Here, we applied the gene expression profiles of 216 samples of LumA, 92 samples of Basal, 122 samples of LumB and 55 samples of Her2. We selected half of the samples of each subtype separately to train the classifier, and the rest of the samples were used to test the classification performance. In addition, the classification performance is compared with the other two key gene selection methods: Information gain and Chi-squared. These two methods are simply based on gene expression data but not considered the mutation data. Due to our method integrated the gene expression data and mutation data, we also compared the classification performance with another integrative method, called Lemon-tree.

To evaluate the performance of the model, we used accuracy measure. Accuracy can be computed as follows:

(1) $$Accuracy=\frac{TP+TN}{TP+FN+FP+TN}$$

where *TP*, *TN*, *FP* and *FN* are true positive, true negative, false positive and false negative respectively. And *F-measure* uses both *Precision* and *Recall* measures to compute the score as follows:

(2) $$F-measure=2*\frac{Precision*Recall}{Precision+Recall}$$

where

(3) $$\begin{array}{c} Precision=\frac{TP}{TP+FP}\\ Recall=\frac{TP}{TP+FN} \end{array}$$

In this study, we used the 10-fold cross-validation to assess the model. As shown in Table 1, Table 2 and Table 3, the accuracy, recall and F-measure of prediction of the our method is up to 98.82%, 0.976 and 0.964 respectively between Basal and other subtypes. For other three methods, the integrative method of Lemon-tree is prior to other two methods, with the accuracy of 98.03%, the recall of 0.928 and the F-measure of 0.939 between Basal and other subtypes. However, the selected driver genes can not successfully classify the subtypes between LumA and other subtypes. The accuracy, recall and F-measure of LumA is 83.13%, 0.801 and 0.812 respectively. It is possible that the selected driver genes have a similar regulation in gene expression.

### 2.4. Topology-Based Pathway Analysis of Identified Driver Genes

To better understand the underlying biological phenomenon, the pathway analysis is used to uncover pathway activity and influence of driver genes. Here, we applied Mirna enrIched paTHway Impact anaLysis (MITHrIL) [32], a technique that extends Draghici et al. [33] and Tarca et al. [34], by combining their effectiveness while improving the reliability of the results. Starting from expression values of genes and/or microRNAs, MITHrIL returns a list of pathway stored according to the degree of their deregulation, together with the corresponding statistical significance (*p*-values), as well as predicted degree of alteration for each endpoint (a pathway node whose alteration, based on current knowledge, affects the phenotype in some way). All terms were first ranked by *p*-value and only the pathways with *p*-value less than 0.01 were selected. The top-18 pathways obtained for breast cancer are shown in Table 4.

As shown in the Table 4, for LumA, the important driver genes are mainly enriched in pathways in Chemokine signaling pathway, HIF-1 signaling pathway, VEGF signaling pathway, Osteoclast differentiation, Cell adhesion molecules (CAMs) and so on after pathway analysis. In Basal, pathways in Natural killer cell mediated cytotoxicity, Chagas disease (American trypanosomiasis), HTLV-I infection are enriched in pathways. In LumB, the pathways analysis are MAPK signaling pathway, PI3K-Akt signaling pathway, Apoptosis, Neurotrophin signaling pathway, Type I diabetes mellitus. In Her2, the main pathways are ErbB signaling pathway, Calcium signaling pathway, HIF-1 signaling pathway, Focal adhesion and Adherens junction after MITHrIL analysis.

### 2.5. Functional Analysis of Driver Genes for Each Subtype

We did Gene Ontology (GO) biological process (BP) and Kyoto Encyclopedia of Genes and Genomes (KEGG) pathways enriched among the subtype driver genes with R package clusterProfiler (http://www.bioconductor.org/packages/release/bioc/html/clusterProfiler.html) [35]. Only the enriched GO terms with *p*-value less than 0.01 and the enriched KEGG pathways with *p*-value less than 0.01 were selected to analyze.

For LumA, the important driver genes are mainly enriched in pathways in Cell adhesion molecules (CAMs), Terpenoid backbone biosynthesis, Thyroid cancer, African trypanosomiasis and so on after KEGG pathway enrichment. With respect to the biological process, nuclear DNA replication, steroid metabolic process, B cell receptor signaling pathway, organic hydroxy compound biosynthetic process are enriched via the GO functional enrichment.

In Basal, pathways in protein digestion and absorption, Lysosome, Natural killer cell mediated cytotoxicity, Apoptosis are enriched in KEGG pathways. In terms of biological process, cholesterol biosynthetic process, secondary alcohol biosynthetic process, multicellular organism catabolic process, multicellular organismal macromolecule metabolic process, steroid biosynthetic process are significantly enriched in GO functional enrichment.

In LumB, the pathways after KEGG enrichment are apoptosis, African trypanosomiasis, NOD-like receptor signaling pathway, Allograft rejection, Graft-versus-host disease. In terms of biological process in GO functional enrichment, driver genes are enriched in response to positive regulation of peptidase activity, activation of innate immune response, positive regulation of innate immune response, cellular chloride ion homeostasis.

In Her2, the main pathways are the HIF-1 signaling pathway, MicroRNAs in cancer, Primary immunodeficiency, Bladder cancer and Pathways in cancer after KEGG enrichment analysis. In terms of biological process, blood vessel morphogenesis, regulation of T cell proliferation, regulation of microtubule-based process, T cell proliferation and regulation of mononuclear cell proliferation are enriched in GO functional enrichment.

## 3. Discussion

In this work, we introduced a module-based framework by integrating transcriptome and genomic data to identify significant driver genes in breast cancer subtypes. By virtue of the consideration the differential expression of genes, a subset of gene whose aberration/expression profile were significantly different between two subtypes may be selected. Also, we constructed a module network by integrating multi-genomic data to identify the specific driver genes.

Because breast cancer data is high heterogeneous and the conventional method for analyzing heterogeneous data perform poorly, we applied this method to the challenging problem of identifying driver genes of breast cancer subtypes. The final result demonstrated that the power of integrative analysis can generate a biological meaningful short list of genes as subtype-specific driver genes.

Furthermore, there are also some limitations of our method. Firstly, in computational and statistical analysis, the method is computationally expensive due to iteration in training the model and searching for the best model. In additional, because the method is based on the statistical machine-learning, it will work better if there are more samples. If there are a few samples in each condition, this method will not perform well. In a word, we developed a novel integrated method based on statistical machine-learning analysis to discover the drivers of breast cancer subtypes, and we showed that the method can generate a short list of biologically meaningful genes that can promote the process of biomarkers discovery.

## 4. Materials and Methods

### 4.1. Breast Cancer Patients Materials

We used processed and normalized breast cancer genomic data as given by TCGA. We downloaded gene expression data from the Agilent 244 K Custom Gene Expression platform, CNV data from the Affymatix Genome-Wide Human SNP 6.0 platform. In this data set, genomic data of 825 patients were obtained. We examined the clinical data from these patients to identify the subtype patients with gene expression data and CNV data. We selected 216 Luminal A, 122 Luminal B, 98 Basal-like and 58 HER2-enriched among the 825 patients, for which their gene expression and CNV data were available as well.

### 4.2. Differential Expression Analysis for Data with Intraclass Heterogeneity

The within group variations are extremely high for the heterogeneous data of breast cancer. Conventional methods to select gene subsets perform poorly when applied to these data with high within class heterogeneity. In this manuscript, the approach of *EMD* (Earth mover’s distance) is applied to acquire gene subset [36]. *EMD* is a measure of distance between two distributions that reflects the minimum cost of transforming one distribution into the other. Whereas test statistics generated by standard differential expression approaches reflect the likelihood that the difference of mean expression between two groups is non-zero or reflect the significance of the association between abundance of short reads of the two groups, the *EMD* test statistic reflects the overall difference between two normalized distributions. These two distributions are represented by signatures.

For the differential expression analysis of genomics data, the signatures are data densities computed from gene expression values’ histograms from each class of samples. Given two signatures *P* and *Q* which are represented as: $P=\{\left(p_{1},w_{p1}\right),\ldots ,\left(p_{m},w_{pm}\right)\}$, where $p_{i}$ is the center of the *i*th histogram cell and $w_{pi}$ is the weight of the cell, which represents the frequency of $p_{i}$; and $Q=\{\left(q_{1},w_{q1}\right),\ldots ,\left(q_{n},w_{qn}\right)\}$, where $q_{j}$ is the center of the *j*th histogram cell and $w_{qj}$ is the weight of the cell, which represents the frequency of $q_{j}$. Given *P*, *Q*, and $d_{ij}$ (the Euclidean distance between $p_{i}$ and $q_{j}$), $f_{ij}$ (the optimal flow between $p_{i}$ and $q_{j}$), the *EMD* scores are calculated as follows:

(4) $$EMD\left(P,Q\right)=\frac{\begin{array}{c} \sum_{i=1}^{m}\begin{array}{c} \sum_{j=1}^{n}f_{ij}d_{ij} \end{array} \end{array}}{\begin{array}{c} \sum_{i=1}^{m}\begin{array}{c} \sum_{j=1}^{n}f_{ij} \end{array} \end{array}}.$$

The *q*-value is the permutation-based estimate of the *FDR* (false discovery rate) which is the expected proportion of rejected null hypotheses [37]. To generate the null contribution, we permuted the sample labels and computed the EMD between the permuted classes for each iteration. We performed 1000 iterations and constructed a null distribution by the median of permuted EMDs for each gene to compute the FDRs.

Then, the FDRs are obtained from a range of significance thresholds. Given $M=[m_{1},\ldots ,m_{N}]$, a vector of median of permuted EMDs, and $EMD=[emd_{1},\ldots ,emd_{N}]$, a vector of observed EMDs, the mathematical representation of FDR for gene *j* and significance threshold *i*, $t_{i}$, is defined as follows:

(5) $$FDR_{ji}=\left\{\begin{array}{c} \frac{\sum_{k=1}^{N}I\left(m_{k},t_{i}\right)}{\sum_{k=1}^{N}I\left(emd_{k},t_{i}\right)}\;\;\;if\;emd_{j}\geq t_{i}\\ 1\;\;\;\;\;\;\;\;\;\;\;\;\;\;\;\;\;\;\;\;\;\;\;\;\;\;\;otherwise \end{array}\right.$$

where *I* is the indicator function:

(6) $$I\left(K,t_{i}\right)=\left\{\begin{array}{c} 1\;\;\;if\;K\geq t_{i}\\ 0\;\;\;otherwise \end{array}\right.$$

*N* is the number of genes in the dataset, and $t_{i}^{\prime }s$ are in descending order from *T* to zero with step ▵:{T,T−▵,T−2▵,…,▵,0}. We set ▵ = 0.001 and *T* to the rounded maximum END minus 1 (*T* = 3). Then, the *q*-value for gene *j* is calculated as:

(7) $$q-value_{j}=min\left(FDR_{j}\right).$$

### 4.3. The Selection of Candidate Modulators

The candidate modulators can regulate other genes in their module. We selected these genes as candidate modulators from the list of aberrant genes if their CNVs frequencies is high across the tumors. The mutation frequency of each gene is calculated as follows:

(8) $$fre=\frac{\#Mutgenes}{N}.$$

where #Mutgenes represents the number of mutation genes in across sample, *N* is the total researched samples.

### 4.4. Initial Modules Construction Based on Normal Gamma Score

Innumerable functional studies suggest that driver mutation are expected to alter gene expression of their cognate proteins, their interacting partners, or genes that share the same biochemical pathway. This will lead to a correlated pattern of gene expression in a network of genes associated with a driver mutation. The initial modules construction step establishes an initial pairing between candidate modulators and gene expression modules by associating each target gene with the single modulator gene that fits it best.

First, for each single candidate modulator gene, we use the gene expression values of the amplified/deleted samples to guide the selection of threshold and consider the gene expression of the amplified/deleted samples to represent appropriate high/low expression levels. We use k-means clustering, using *k* = 2 and the normal and amplified/deleted samples as the two initial clusters to fit two normal distributions. The boundary between two clusters is the selected expression threshold level for this candidate modulator gene. The selected threshold is used to split the expression of each target gene.

Then, the expression of each target gene is split into two sets: those in the tumor samples in which the driver’s expression is below the threshold, and those in the tumor samples in which the driver’s expression is above the threshold. We adopted the possible models and used a statistically based likelihood score called the Bayesian score to evaluate a model’s fitness to the data [38]. We use Normal Gamma distribution for our likelihood function. The Normal Gamma scoring function is used to compute the quality of this split, thus measuring a target gene’s fit with a candidate modulator. Given leaf, a vector of the gene expression values of entire samples, and a vector Leaf which represents the split gene expression values contained in the leaf; α and λ are the parameters and *N* is the size of Leaf. The Normal Gamma score is described below:

(9) $$\begin{array}{ll} Score= & -N*\ln\left(\sqrt{2\pi }\right)+\frac{\ln\left(\frac{\lambda }{\lambda +N}\right)}{2}+\ln\left(\Gamma (\alpha^{+})\right)-\\ & ln\left(\Gamma (\alpha )\right)+\alpha *\ln\left(\beta \right)-\alpha^{+}*\ln\left(\beta^{+}\right) \end{array}$$

where,

(10) $$\beta =Max(1,\;\frac{\lambda *(\alpha -2)}{\lambda +1})$$

(11) $$\beta^{+}=\beta +\frac{Var(Leaf)*N}{2}+N*\lambda *\frac{\bar{Leaf^{2}}}{2*(N+\lambda )}$$

(12) $$\alpha^{+}=\alpha +\frac{N}{2}$$

A split is scored by comparing the score of the split data to the score without split, along with a penalty for the split. After the score is computed for all pair-wise combinations of candidate modulators and target genes, each gene is assigned to the single highest scoring candidate modulator.

### 4.5. Module-Network Learning

**Figure 4:**
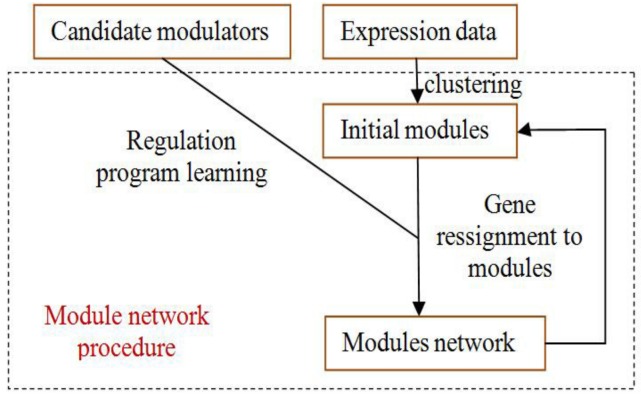
The process of module-network learning. It is an iterative procedure that determines both the partition of genes to modules and the regulation program for each module.

The module-network learning step uses the modules generated by the initial modules step as a starting point and uses an iterative approach to improve the score of the modules and their regulation programs. In each iteration, the procedure learns a regulation program for each module and re-assigns each gene to the module whose program best predicts its behavior. The learning process is shown in Figure 4.

In our iterative learning procedure, the Expectation Maximization (EM) algorithm [39] is applied to search for the model with the highest score. Normal Gamma gives a higher score to data with lower variance and hence finds splits that create two different contexts that represent two distinct behaviors. In the procedure of learning, we also use Normal Gamma distribution for our likelihood function. We adopted the strategy in [38]. The likelihood function as follows:

(13) $$L\left(M:D\right)=P\left(D|M\right)=\prod_{m=1}^{M}\left(P\left(x\left[m\right]|\tau ,A\right)\right).$$

The *M* is a triple (C,τ,A), where *C* is a module set, τ is a module network template for *C*, and *A* is a module assignment function for *C*. Given the training set $D=\{x_{1},\ldots ,x_{M}\}$, which consisting of *M* instances drawn independently from an unknown distribution *P*(*X*). We assume that the set of modules *C* is given, and we wish to estimate this distribution using a module network over *C*.

The procedure of EM algorithm consists two steps: an E-step and an M-step. These two steps are iterated until that fewer than 10% of the target genes have been re-assigned to a different module.

(i) In the M-step, the procedure is given a partition of the genes into modules and learns the best regulation program for each module. For computational efficiency, some M-step optimize only the parameters and leave the regulation program structure unchanged. We recursively learned the regulation choosing, at each point, the candidate modulator that best splits the gene expression of the module genes into two distinct behaviors. All candidate modulators and split values were evaluated and the modulator-split combination that achieves the highest improvement in score is selected.
(ii) In the E-step, given the inferred regulation programs, we determined the module whose associated regulation program best predicts each gene’s behavior. Specifically, we iterated over all genes, one at a time, and moved each gene into the module which provides the highest improvement in the score. This step is guaranteed to improve the score, or leave it the same.

### 4.6. The Identification of Candidate Driver Genes

**Figure 5:**
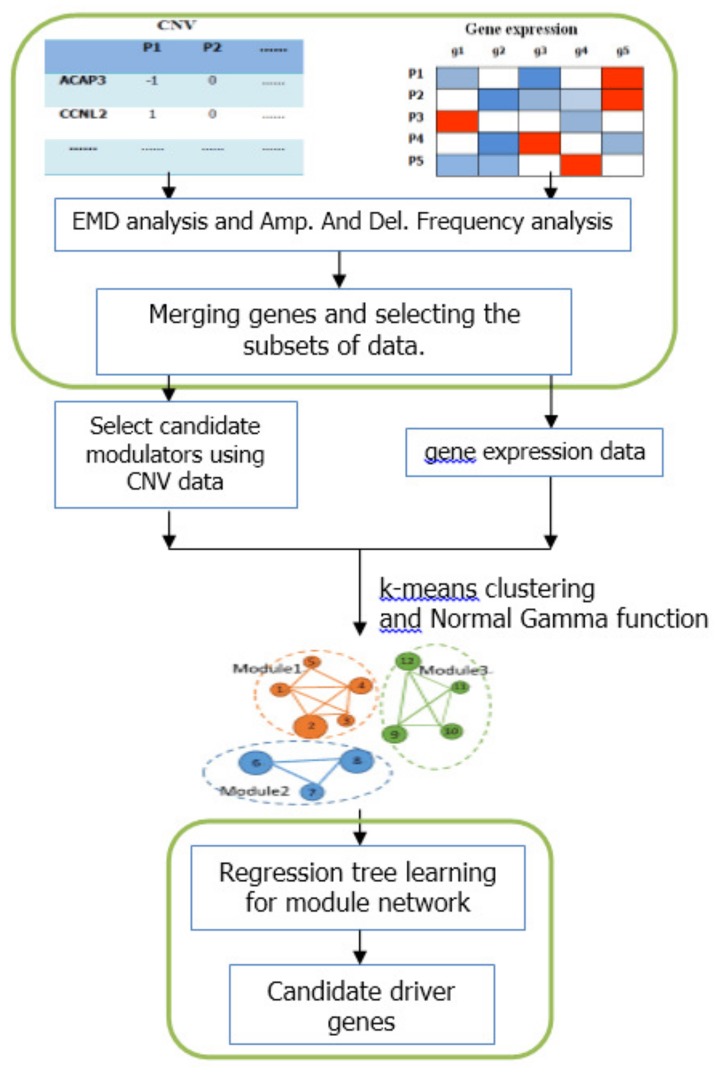
Schematic diagram of the integrative method based on module-network. The first part indicates the pre-processing steps based on the differential expression analysis. The middle part is the construction of the initial modules. The bottom part represents the process of module network learning.

We proposed an integrative frame based on module-network to identify candidate driver genes. The framework process is shown in Figure 5. First the EMD difference analysis and frequency analysis were used to select subset of data. Then the initial modules were constructed by *k*-means clustering and Normal Gamma scoring function. And the module-network learning was used to obtain the final modules and candidate modulators. The module-network learning step was run *N* times. We calculated the frequency of appearance of each candidate modulators as follows:

(14) $$fre\_app=\frac{\#times\;of\;appearance}{N}.$$

where #timesofappearance is the appearance times of each modulators in *N* runs. we filtered the set of candidate modulators and left only genes that appeared in at least one regulation program in at least 40% of the runs. The final run of the module-network learning algorithm was run using the filtered set of candidate modulators and these modulators were considered as candidate driver genes. The whole method is shown as Algorithm 1.

**Algorithm 1** Integrative model based on module-network for cancer subtypes**Input**: CNV data and gene expression data of two subtypes**Output**: A short list of gene sets **The 1th step:** Difference analysis EMDSort $\left(P,Q,f_{ij},d_{ij}\right)$  (a) compute the EMD(P,Q) using $f_{ij}$ and $d_{ij}$ according to the Formula (4). $f_{ij}$ is the flow and the $d_{ij}$ is the Euclidean distance.  (b) compute the $FDR_{ji}$ according to the emd-values.  (c) compute the *q*-value according to the $FDR_{ji}$. **The 2th step:** Initial modules construction  (a) fit two normal contributions by *k*-means clustering and select the threshold *T* for each modulator.  (b) split the expression of the target gene into two sets (A,B) according to the threshold *T*.  (c) Given a leaf vector leaf, the parameters α and λ, the size of Leaf
*N*.  (d) compute the Score(target_gene,modulator) of the split using the Formula (9).  (e) assign the target gene into the single highest scoring candidate modulator. **The 3th step:** Module network learning **repeat**  (a) search for a regulation program for each module.  (b) reassign each gene to the module whose program best predicts its behavior.  (c) compute the proportion of re-assigned genes pro. **until** (pro<0.1) **The 4th step:** The identification of candidate driver genes.

## Figures and Tables

**Table 1:** The accuracy of 10-fold cross validation between subtypes.

Subtypes	Module-Network	Information Gain	Chi-Squared	Lemon-Tree
LumA-others	83.13%	79.60%	80.39%	85.88%
LumB-others	84.70%	76.47%	76.86%	80.39%
Basal-others	98.82%	97.64%	98.82%	98.03%
Her2-others	92.94%	92.94%	94.90%	92.15%

**Table 2:** The recall of 10-fold cross validation between subtypes.

Subtypes	Module-Network	Information Gain	Chi-Squared	Lemon-Tree
LumA-others	0.801	0.844	0.853	0.827
LumB-others	0.847	0.402	0.291	0.5
Basal-others	0.988	0.952	0.976	0.928
Her2-others	0.929	0.56	0.6	0.44

**Table 3:** The F-measure of 10-fold cross validation between subtypes.

Subtypes	Module-Network	Information Gain	Chi-Squared	Lemon-Tree
LumA-others	0.812	0.790	0.798	0.842
LumB-others	0.719	0.491	0.415	0.590
Basal-others	0.964	0.930	0.964	0.939
Her2-others	0.590	0.608	0.697	0.523

**Table 4:** Top-18 pathways obtained for breast cancer subtypes after enrichment performed by MITHrIL.

LumA		LumB	
**Pathway**	**p-Value**	**Pathway**	**p-Value**
Chemokine signaling pathway	0	MAPK signaling pathway	0
HIF-1 signaling pathway	0	PI3K-Akt signaling pathway	0
VEGF signaling pathway	0	Apoptosis	0
Osteoclast differentiation	0	Neurotrophin signaling pathway	0
Hippo signaling pathway	0	Type I diabetes mellitus	0
**Her2**		**Basal**	
**Pathway**	**p-Value**	**Pathway**	**p-Value**
ErbB signaling pathway	0	Natural killer cell mediated cytotoxicity	0
Calcium signaling pathway	0	Chagas disease (American trypanosomiasis)	0
HIF-1 signaling pathway	0	HTLV-I infection	0
Focal adhesion	0		
Adherens junction	0		

## Supplementary Materials

The main code is available online.
