# Supplementary material for: The Integrative Method Based on the Module-Network for Identifying Driver Genes in Cancer Subtypes
Source: Molecules. 2018 Jan 24;23(2):183. doi: 10.3390/molecules23020183 (PMC6099653; doi:10.3390/molecules23020183)
Supplement: Supplementary file 1 [file molecules-23-00183-s001.pdf]

The main code is provided in "https://github.com/lixing000/Module-Net".
